# Brain-inspired energy efficient technologies for next-generation artificial intelligence

**DOI:** 10.1007/s00422-026-01038-4

**Published:** 2026-02-23

**Authors:** Hillel J. Chiel, Jay S. Coggan, Gourav Datta, Jean-Marc Fellous, William R. P. Nourse, Roger D. Quinn, Peter J. Thomas

**Affiliations:** 1 Department of Biology, Case Western Reserve University, Cleveland, USA; 2 Department of Biomedical Engineering, Case Western Reserve University, Cleveland, USA; 3 Department of Neurosciences, Case Western Reserve University, Cleveland, USA; 4 NeuroLinx Research Institute, La Jolla, USA; 5 Department of Electrical, Computer, and Systems Engineering, Case Western Reserve University, Cleveland, USA; 6 Institute for Neural Computation, University of California San Diego, La Jolla, USA; 7 Department of Mechanical Engineering, Case Western Reserve University, Cleveland, USA; 8 Department of Mathematics, Applied Mathematics, and Statistics, Case Western Reserve University, Cleveland, USA; 9 Department of Cognitive Science, Case Western Reserve University, Cleveland, USA

**Keywords:** Neuroscience, Artificial intelligence, Energy efficiency, BioFlop

## Abstract

Since the advent of widely accessible AI tools, AI technology has been in high demand by businesses, academic researchers and individuals. Technology companies are building AI infrastructure at a rapid pace, and these facilities consume vast and growing resources, particularly electricity and water, with significant real and projected climate impacts. There is a need for new research initiatives to support long time horizon efforts to develop energy efficient computing capabilities to support the continued growth of AI infrastructure in a sustainable fashion. Such efficiency is required at both the hardware and software levels. *Where can industry turn for examples of ultra-low power, energy efficient computing?* We argue here that neurobiological principles offer rich and under-exploited sources of inspiration for energy efficient NeuroAI, and that new partnerships between industry and academia should be developed in this direction.

## Introduction

One way to achieve energy efficient artificial intelligence (AI) is to study how nature generates naturally intelligent behavior that minimizes energy expenditure. All biological organisms are shaped by developmental and evolutionary processes that require energy for their survival and reproduction. An organism whose energetic costs chronically exceed its energy intake will suffer dire consequences, and on the evolutionary time scale, will be eliminated. We will briefly review energy usage in biological brains and bodies and then suggest potential new directions for artificial intelligence in general and NeuroAI in particular, a new type of artificial intelligence designs inspired by brain mechanisms. The ideas presented could also apply to robotics and autonomous agents.

## Biological brains and bodies

### Biological brains and energy

**Fig. 1:**
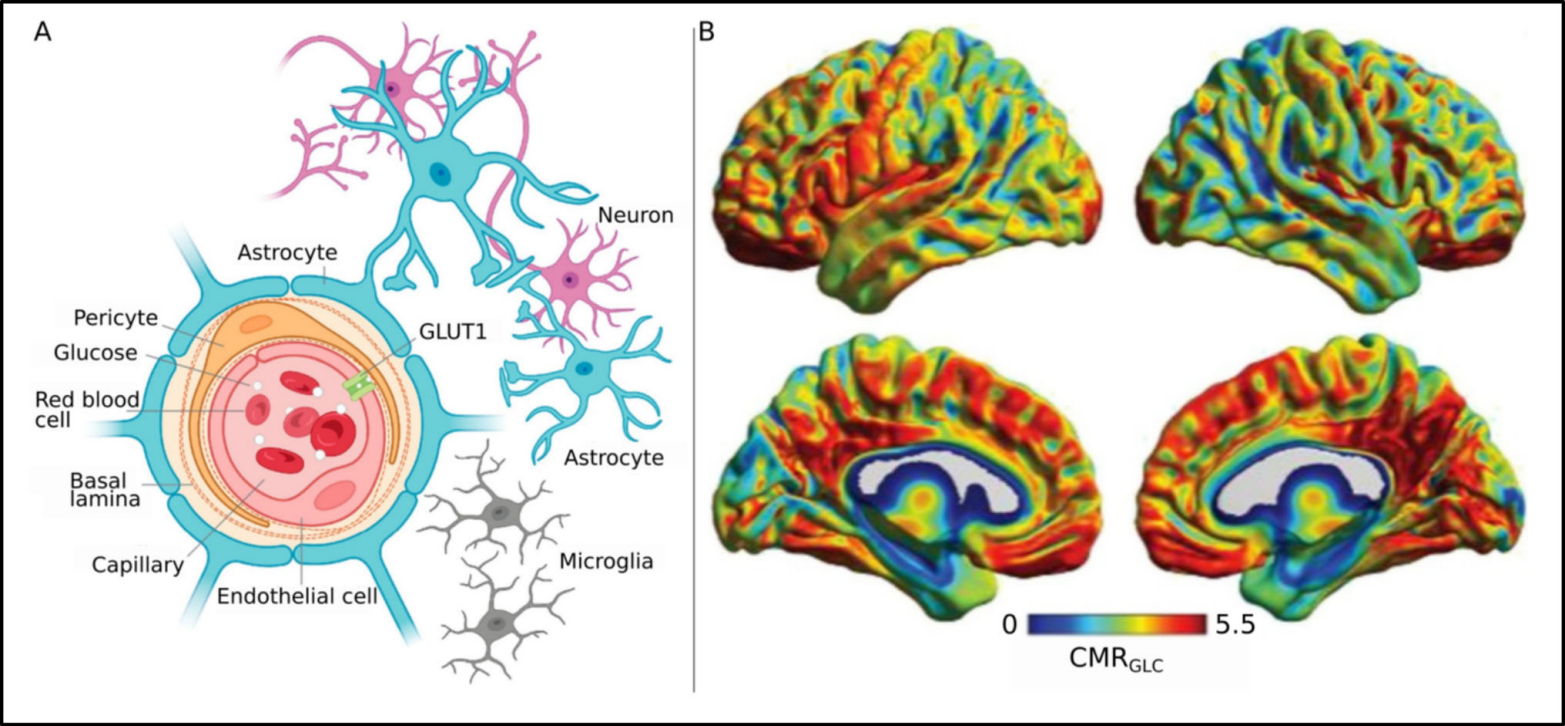
Energy usage in the brain. **A**. Glucose crosses the blood-brain barrier, and is metabolized by neurons, astrocytes, and other glial cells. **B**. Glucose utilization can be measured, creating an intensity map of activity throughout the brain. Images (used with permission) from (Jamadar et al. 2025)

Biological brains (Fig. 1) are by far the most energy efficient computing devices that we know, using only about 20 W of power in the case of the human brain, which is more than 1 million-fold less than the world’s largest supercomputer, a machine that does not yet approximate human intelligence, in spite of the impressive feats of the best LLMs (large language models) today. As Moravec articulated, tasks that are difficult for humans, such as playing chess or learning languages, are easy for AI, while many tasks that are easy for humans are extremely difficult for machines (Moravec 1988). This points to many important issues that are beyond the scope of this perspective. But as a matter of example, self-driving car enthusiasts were premature and underestimated what the human brain needs to do to drive to work without wreaking havoc. Some of what humans do best, and easily, are among the hardest and most important things they do for survival. The dynamical thinking at which humans excel, and that is at the core of biological intelligence, is not even attempted in LLM or any other AI model.

It is possible that we will not know exactly how efficient the brain actually is in units of Watts/Flops until we establish a biological equivalent, such as the BioFlop, as coined by (Stiefel and Coggan 2023). This hypothetical value may be difficult to pin down precisely due to the analog nature of biological computing. In this same paper, the authors introduced a measure of AI efficiency; the ERASI equation. This calculation has initially shown that the cost of mimicking the human brain (based on what we currently know about its computations) would be orders of magnitude higher than the entire annual US energy output. As a benchmark, the authors used estimates of energy use from the now disbanded Blue Brain Project of EPFL (https://bluebrain.epfl.ch/bbp/research/domains/bluebrain/), a Swiss government initiative to simulate an entire mouse brain based on a biologically realistic reconstruction of brain circuits. This initiative has birthed a very different approach to AI than LLMs, one that attempts to discover new biological learning rules that could perhaps be implemented in future Machine Learning (ML) systems (see also https://www.openbraininstitute.org; https://www.inait.ai). Currently, these efforts and other similar AI work inspired by the brain are captured by the generic term “NeuroAI” (Zador et al. 2023; Sadeh and Clopath 2025; Arbib 2025).

Artificial neural networks were initially developed based on theories in neuroscience from the 1950s, but neuroscience has made a lot of progress since then, which has not been incorporated into mainstream AI and ML. For example, one of the many limitations of LLMs and other Artificial Neural Network approaches is underestimating how single cells process information. While LLMs use a “neural” architecture, their low-level components have little in common with the physiology of nerve cells and biological synapses, which are themselves capable of processing and transforming information. Artificial neural networks are abstracted from synaptic integration and all-or-none action potential generation present in biological networks, to a simplified linear summation followed by a nonlinear response such as a logistic, tanh or ReLu operator^1^ (Haykin 1999). There is a growing theoretical base for conceptualizing this level of computation (Rieke et al. 1997; Poirazi and Mel 2001; Arcas et al. 2003; Coggan et al. 2020; Lillicrap et al. 2020; Coggan et al. 2022). There may be more fundamental and yet unexplored units of computations than the neuron (e.g. dendrite, synapse, ion channels or metabolic states), which could also point in new directions for energy efficiency (Boahen 2022). In short, there may be great value in synergistically coupling brain research and new initiatives in AI research. Beyond biomimicry, we have much more to learn from biological solutions and computational neuroscience about the fundamental mechanisms of intelligence. It cannot be overemphasized that basic computational and experimental neurobiology research will lead to more powerful and efficient AI.

Here we review concepts from biology and use them as a springboard to suggest examples of areas where AI technologists might benefit from deeper understanding of neurobiology and behavior.

### Biological neural dynamics

Biological neurons use energy to maintain their ability to process and generate both electrical and chemical signals. Across the membranes of nerve cells, energy requiring pumps (e.g. the sodium/potassium ATPase pump) ensure that, inside the cell, potassium ion concentrations are higher and sodium ion concentrations are lower than outside the cell. The differential concentrations of ions across the cell membrane are the basis for the *resting potential*, a nonzero membrane potential difference (often about -60 mV) that allows neurons to rapidly respond to and integrate electrical inputs (Berndt and Holzhutter 2013). Neurons also maintain a complex panoply of *ion channels* that can be *gated* (i.e., can be opened or closed) by voltage, specific chemicals that bind to specific receptors, the calcium ion, second messengers, or by different forms of energy (e.g., photons, temperature gradients, mechanical deformation, applied force, or vibration) (Bhattacharjee 2023). The specific complement of ion channels in a neuron’s membrane endows it with complex dynamical properties, so that neurons can be silent, or spontaneously fire *action potentials*. Some neurons can generate spontaneous rhythmic bursting (pacemaker neurons) (Ramirez et al. 2004), and neurons can have multiple stable dynamical properties (e.g., they can be switched from being silent to bursting regularly) (Connors and Gutnick 1990). Unlike most current NeuroAI systems where all neuronal elements are identical, biological neurons exhibit a high degree of individual differences that may be at the origin of high computational capacity and efficiency. Ion channels responsive to energy and chemicals in the environment allow neurons to sense environmental conditions and are the basis for the sensations of touch (Jin et al. 2020), taste (Spector and Travers 2021), smell (Fulton et al. 2024), sight (Barret et al. 2022), hearing (Zheng and Holt 2021), and the ability to sense the location, position and overall internal state of the body itself (proprioception; (Moon et al. 2021)). In the cognitive areas of the brain (involved in learning, memory, and decision making), neurons are mostly silent and communicate with each other either using bursts of action potentials (packets of information, temporal summation) or synchronized action potentials (simultaneous production of tightly time-locked action potentials to a target neuron, spatial summation) (Tiesinga et al. 2008; Lisman 1997). Again, these rich intrinsic firing properties and dependences of firing on the environments are usually not present in NeuroAI systems.

### Biological neural synapses

Neurons connect to one another via *synapses*, which may be either electrical or chemical, and the energetic requirements of synaptic activity are one of the largest uses of energy by neurons (Li and Sheng 2022; Harris et al. 2012). Electrical synapses provide a way of rapidly linking activity between neurons, whereas chemical synapses provide somewhat slower but highly modifiable means of exciting or inhibiting another neuron, potentially greatly amplifying or dampening the strength of the signal. Chemical synapses can connect neurons over multiple temporal and spatial scales: the rapid binding of neurotransmitters released within the synaptic cleft can induce rapid changes in other neurons, with some receptors responding more slowly than others. Moreover, some neurotransmitters can diffuse out of the specialized synaptic endings and bind distant receptors, affecting neurons that are not directly connected over a much larger spatial volume. *Neuromodulatory* transmitters have been shown to lead to higher energy consumption during more complex cognitive processing in the cerebral cortex (Castrillon et al. 2023). Maintaining chemical synaptic *transmission* requires significant energy, and so does synaptic *plasticity*, the change in synaptic strength as a function of conjunctive activity of a neuron and other connected neurons. There are two kinds of plasticity: the first is short term (depression, facilitation) in the order of 10-100ms and controls the way sequences of signals are transferred from neuron to neuron (e.g. frequency filtering) (Asopa and Bhalla 2023; Yu et al. 2025b). The second is long term (LTP - long-term potentiation, LTD - long-term depression, minutes to days) and controls the “relevance” of the synapse to general neural computation (Murai and Goto 2025; Stanton 1996; Bear and Malenka 1994). This type of plasticity has inspired “changes in synaptic weight” rules in artificial neural networks, while the first short-term type is generally ignored. Another important energy requirement is the *remodeling* of synaptic connections, as neurons may grow additional connections to strengthen or remove connections to weaken interactions with other neurons. In cognitive areas, a given neuron receives potential inputs from about 10,000 other neurons, and a fraction of these inputs are almost always active, keeping the neurons in a constant state of subthreshold fluctuations, ready to fire in response to small but significant input signals. This type of background subthreshold activity is not yet accounted for and used by NeuroAI systems.

### Biological neural shapes and efficient computation

Neurons have complex shapes (*morphology*) that allow them to process inputs both spatially and temporally, and studies suggest that energy is crucial for determining and maintaining their shapes (Wen and Chklovskii 2008). Early anatomists saw the similarity between neuronal branching and those of trees, referring to the fine branches of neurons on which connections are made as dendritic arborizations (Yuste and Tank 1996). Neuronal shapes determine whether sub-threshold electrical inputs travel to the spike-initiating zone and generate outputs from the entire neuron. Furthermore, localized interactions can allow neurons to process inputs at many different locations in parallel. Energy requirements influence the maintenance and changes in shape that neurons undergo over an animal’s lifetime (Rumpf et al. 2023). Artificial Neural Networks usually consider neurons as shapeless ‘point-like’ units.

### Maintaining biological neural networks

Another major use of energy in the brain is the maintenance of the neuronal support cells, the *glial cells*, which play significant roles in providing energy to neurons, and for generating the wrapping material (myelin, which gives white matter its name) that allows rapid transmission along the long axons of neurons. White matter is responsible for long distance communication between regions of gray matter, which contain the dendrites, cell bodies, and synapses of the neurons. White matter consumes energy at a much lower rate than does gray matter (Yu et al. 2018). Glial cells also regulate the amount of energy available to nerve cells (Shoenhard and Sehgal 2025) and play a critical role in clearing debris and responding to attacks from bacteria, viruses, and other sources of inflammation, which also requires significant energy (Jamadar et al. 2025). There are no implementations of glial cells in NeuroAI system,

### Biological neural architectures and sparse connectivity

**Fig. 2:**
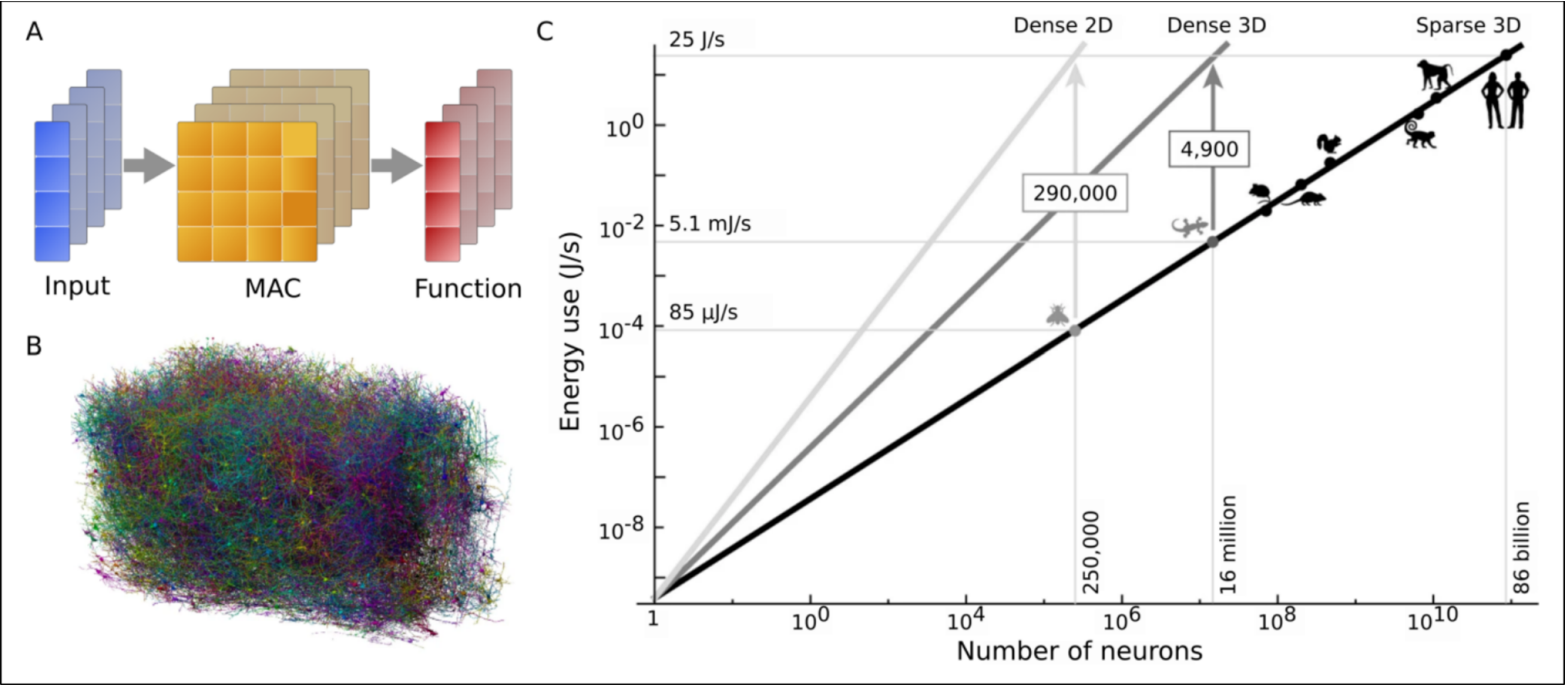
Sparsity and structure in natural and artificial neural networks.** A**. In modern AI systems, most computation is performed on hardware (GPU, NPU) which uses dense vector and matrix multiplication via multiply-accumulate (MAC) modules. As a toy example, multiple input vectors are multiplied by a weight matrix, then the resulting vectors are processed by some activation function. These operations can be carried out in parallel, but this dense MAC operation is at the core. **B**. In contrast, natural networks of neurons are interconnected in 3D space, with sparse connectivity and activity patterns. Image (used with permission) from (Gamlin et al. 2025). **C**. Energy use in dense 2D accelerators such as GPUs/NPUs scales quadratically with increasing numbers of neurons. Biological systems, on the other hand, scale linearly in energy use. Image (used with permission) from (Boahen 2022)

Across phylogeny, different regions of the nervous system are organized into different complex *architectures*. The central complex of arthropods, the vertical lobe system of the octopus, the hippocampus, the cerebral and cerebellar cortices in primates and humans are all striking examples of highly organized anatomical organizations of input, output and interneuronal circuitry (Luo 2021). Neural architectures clearly subserve specific functions. In addition to highly organized local patterns of connectivity, there are long-range connection tracts, many of which constitute interconnection “hubs”, which have the highest energy requirements (Ceballos et al. 2025; Jamadar et al. 2025). Neuronal architectures are generated through *developmental* processes, which also require and are regulated by energy availability (Ghosh et al. 2023). Figure 2 illustrates some consequences of sparsity and structure in natural versus artificial neural networks. More needs to be done in current NeuroAI system to allow for and exploit energy-dependent ‘developmental-like’ changes in architectures.

### Transmission rates versus information transfer in biological nervous systems

Patterns of neural activity achieve a tradeoff between frequency of transmission and information transfer because of energetic constraints. Although neurons are capable of high tonic firing rates of firing, the energetic costs of operating action potentials (and especially synapses) favor lower rates of transmission. Ongoing spontaneous activity within the brain provides a floor to the minimum rates of transmission that are above the background noise, but also may provide the brain with the ability to mobilize long range connectivity much more rapidly and with relatively small energy expenditures (Harris et al. 2012). Interestingly, it has been suggested that transient patterns of high firing rate (bursts) could carry more information than single action potentials (Lisman 1997), and that bursting may be key to implementing classic artificial neural network mechanisms such as back-propagation (Sun et al. 2021; Payeur et al. 2021)

### Biological neural network preprocessing

A major role of nervous systems is to filter out irrelevant stimuli and enhance stimuli that are most likely to be immediately relevant. Processes such as habituation act to reduce activity in sensory neurons that would otherwise be activated by irrelevant stimuli (Ramaswami 2014). Feedforward activation makes it easier to begin to excite neurons that are most likely to be encountering sensory inputs or generating motor outputs next (Briggs 2020). These type of preprocessing increases the effectiveness and efficiency of information processing.

### Biological brains and behavior

Unless an organism can photosynthesize and reproduce asexually, it must be able to move through and interact with its surrounding environment to obtain food and mates, and to avoid predators. Within an organism, internal organs, such as the heart, lungs, and digestive system, also need to be controlled. Thus, neurons must interact, through the *neuromuscular junction*, with smooth, cardiac and skeletal muscle. Once again, energy plays critical roles in both the function of muscle and in the behavior that it generates. Depending on the size and speed of a behavior, energy may be partitioned among inertial energy, viscous (dissipative) energy, elastic energy, and responses to gravity (Sutton et al. 2023). These different forms of energy imply different stabilities in response to perturbations, which in turn demand different controls strategies for behavior. Energy obtained through feeding must also be partitioned between the needs of the body and of the brain, and complex regulatory mechanisms are responsible for maintaining the appropriate levels of energy for each (Myers et al. 2021). Such energy-expenditure specializations would likely benefit autonomous mobile or actuating artificial systems such as NeuroAI-enabled robots.

### Unsupervised learning in biological nervous systems

Over longer periods of time, behavior is regulated by positive and negative reinforcement from the environment (Sutton and Barto 2018). One component of these regulators is the physical effort and energy that must be expended to achieve goals (Jiang et al. 2024). Another is the experience of pain or pleasure in response to an organism’s actions, and emotions that reflect an organisms overall internal state and response to past, present and future events (Fellous and Arbib 2005; Arbib and Fellous 2004). Artificial neural networks are trained and modified using datasets in which elements are considered intrinsically equally important. Their relative importance is determined by how often they occur, rather than by how relevant they might be to learning a particular body of knowledge. AI systems typically do not learn in “one shot”, as humans and animals often do (Yu et al. 2025a). More generally, living organisms are autopoietic, networks of processes that maintain and renew themselves through their own activity (Maturana and Varela 1980). Reducing the amount of training data, using considerations of their intrinsic value to learning could yield significant improvement in the explainability of AI algorithms (Wells and Bednarz 2021) and energy consumption savings in future NeuroAI learning systems.

## Biologically-inspired energetically efficient approaches to AI

Given these general insights from biological systems, we next list some ideas that could be explored in the near-term to improve the energy efficiency of AI systems.

### Neuromorphic computation

New approaches to computing that are inspired by biological neurons may lead to significant savings in energy expenditure. Hardware approaches subdivide into attempts to capture the complex analog processing that occurs in biological neurons using very low energy levels; others attempts to use standard silicon technology to emulate neurons, and some commercially available neuromorphic chips can be used for rapid preprocessing of sensory information on chip (e.g., neuromorphic cameras); others use sparse connectivity and computation for high energy efficiency (e.g., BrainChip, SpikeCore, Brain Corp). Intel has developed a neuromorphic chip (Loihi2) that uses a spiking neural network for computation, and that communicates and computes using “spike events”, reducing power consumption. In contrast to this chip, which ensures that all processing is completed at each step, a more distributed architecture has been pioneered by SpiNNcloud, which has multiple cores that simulate neurons communication asynchronously. By using denser local connectivity (but not full connectivity) and sparser global connectivity, these chips can also be more energy efficient. Many neuromorphic chips also incorporate plastic synapses that allow for learning.

### Sparse representations may be energy efficient

There is a large body of neurobiological and computational evidence pointing to sparse coding in the nervous system (Beyeler et al. 2019). Sparse representations that improve reconstruction from noisy data have been intensively studied in applied mathematics (Calvetti et al. 2019; Calvetti et al. 2020; Calvetti and Somersalo 2024). The connection between sparse representation and energy efficiency has been investigated in neurobiology (Hu et al. 2012; Sacramento et al. 2015; Moosavi et al. 2024). In addition to saving energy, sparse representations could also render AI systems more robust to adversarial attacks and catastrophic failures.

### Sparse bursting activity is common in motor control/central pattern generator systems

**Fig. 3:**
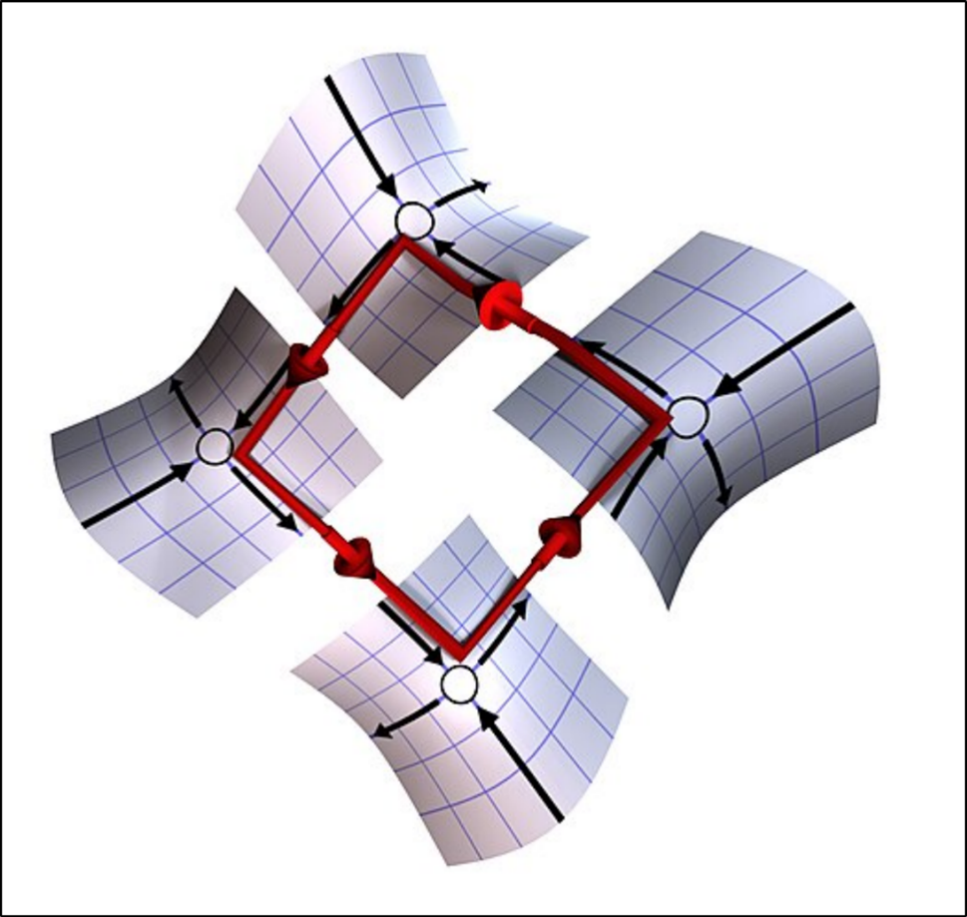
Diagram illustrating a heteroclinic channel. A heteroclinic cycle in a dynamical system is a sequence of trajectories connecting the flow out of and into successive saddle point equilibria. A stable heteroclinic channel attracts all nearby trajectories. See (Rouse and Daltorio 2021) for a detailed description. Image (used with permission) from (https://en.wikipedia.org/wiki/Heteroclinic_channels)

In neuro-motor systems, it is inefficient to simultaneously tense opposing muscles, or to activate muscles that are not needed for a given movement. Thus, it is no surprise that patterns of activity in biological motor control systems exhibit sparse bursting patterns, in which a small number of neurons are active at any one time. Such sparse activity patterns are mediated by inhibition, in which the activity of one cell suppresses the activities of others. Similarly, inhibition plays a key role in winner-take-all (WTA) algorithms that select one out of many possible answers (Maass 2000). If a unit consumes more energy when it is “active” than when it is “silent” then WTA dynamics lends itself to energy-efficient implementation. Inhibition also underlies stable heteroclinic channels (SHCs: Fig. 3) that have been proposed as a dynamical architecture supporting sparse, functionally effective activation patterns in motor systems (Shaw et al. 2015; Horchler et al. 2015; Rouse and Daltorio 2021; Mengers et al. 2025), sensory systems (Laurent et al. 2001; Rabinovich et al. 2010) and cognitive processing (Afraimovich et al. 2011; Rabinovich and Varona 2018). Research initiatives to study SHCs, WTA architectures, and other mechanisms for inhibition-dominated connectivity in AI systems may lead to novel energy-efficient solutions.

### Neuromodulation suppresses the activity of subnetworks that are not needed for a given task

In motor control systems, metabolic resources are redirected to muscle systems that are actively being used. Switching muscles “on” and “off” is regulated by neuromodulation (Sillar et al. 2014). Similarly, activity in neural subsystems is also regulated by neuromodulation. Specializing circuits for specific computational tasks and then powering down those circuits when those tasks are not needed may recapitulate biological-like (biomorphic) efficiencies. More generally, the extent to which neuromodulation in general (e.g. via serotonin, norepinephrine, dopamine, and others) has evolved to improve the efficiency of neural computation (rather than perform neural computation *per se*) is understudied and deserves attention (Castrillon et al. 2023; Yu et al. 2025c). Implementing neuromodulatory principles in “classic” AI or more modern NeuroAI models might significantly improve their performance, and consequently, their energy expenditure.

### Plasticity modulation for adaptive intelligence

In addition to gating circuit activity for energy efficiency, neuromodulation in biological systems plays a central role in regulating when and where learning occurs. Neuromodulators such as dopamine, acetylcholine, and norepinephrine do not directly trigger but instead modulate synaptic plasticity, guiding long-term changes in neural circuits based on behavioral relevance, reward prediction, or uncertainty (Marder 2012). This dynamic plasticity control enables animals to learn selectively, preserving stable functions while adapting rapidly to new situations—a capability that current AI systems, including LLMs, largely lack. Instead, these models rely on static, global learning schedules and costly retraining for adaptation (Bommasani et al. 2021). By integrating neuromodulatory principles, such as plasticity gating conditioned on internal goals or novelty signals, future AI systems could enable targeted, context-aware updates to their internal states. This approach offers a path to learning-to-learn (meta-learning) in large-scale models, improving data efficiency, stability, and the ability to generalize across tasks, while avoiding the overhead of continual full-network retraining (Wang et al. 2016; Wang et al. 2018; Hattori et al. 2023; Goudar et al. 2023).

### Memory changes with time and use, yielding more efficient representations

Unlike computers, we (humans and animals alike) store information neither “forever” nor exactly as it was acquired. We do not store pixel-level images, or second-by-second sequences of speech or episodic memories. We have an ability to abstract, simplify or ignore our inputs as they come in, and to re-shape our memories as a function of how or how often we use them, or as a function of their intrinsic “importance”. These features have undoubtedly evolved (at a cost for reliability) to address our limited capacities to perceive and memorize, saving energy and time. AI systems do not usually implement the (useful) mechanisms of forgetting, or memory consolidation (as during sleep (Rouast and Schonauer 2023)). While industry considers these features as deleterious and artifactual, to be avoided and corrected to achieve reliable and precise recall, research into LSTM networks with “forgetting states” has shown promise for continuous learning and other applications (Gers et al. 2000; Wang et al. 2025). New research should explore the extent to which a trade-off can or should be achieved between full recall and precision, and efficiency of representations.

### Neuroscience-inspired memory efficiency

Biological brains achieve remarkable memory efficiency through mechanisms such as sparse coding, synaptic consolidation, and multi-scale memory hierarchies. Unlike large-scale AI models that rely on dense parameter storage and exhaustive training data exposure, the brain encodes information using compact, context-dependent neural activations, often reusing the same networks across tasks via dynamic routing or population coding. Furthermore, systems consolidation, involving transfer from fast-learning hippocampal circuits to slower cortical storage, enables long-term retention without continuous memory access. These strategies contrast with the monolithic memory structures of LLMs, which grow in size and cost with increasing data. Incorporating such biological insights—e.g., sparsity-inducing priors, gated memory consolidation, or attention-modulated storage and retrieval mechanisms—could significantly reduce the memory footprint of large models without sacrificing performance. This approach not only improves energy and storage efficiency, but also supports more flexible, lifelong learning paradigms where memory is treated as a dynamic, structured resource rather than a static archive (Olshausen and Field 1996; Zenke and Gerstner 2017; McClelland et al. 1995).

### Multi-resolution representations may be energy efficient

Receptive fields such as those found in visual cortex have inspired significant contributions in Artificial Learning systems, such as convolutional neural networks (Celeghin et al. 2023). Typically, in these systems, all convolutional kernels are similar. Not all information, however, needs to be represented at the same resolution. The visual system has evolved multi-scale coding (different receptive field sizes within cortical areas, e.g. cortical area V1, and across areas, e.g. cortical areas V1-V4-IT). Spatial navigation in large environments uses multi-scale hippocampal place fields (Harland et al. 2021; Eliav et al. 2021). This multi-scale representation has been shown to have many computational advantages, including efficiency and fast attentional shifting. Most AI systems do not make use of multi-scale representations.

### Cortical traveling waves and structured dynamics in large-scale neural models

A growing body of neuroscience research points to the central role of spatiotemporal cortical waves, such as alpha, beta, and theta oscillations, in orchestrating perception, attention, and memory in the brain. These waves enable multiplexed signaling and efficient integration across distributed cortical areas by rhythmically modulating neuronal excitability (Muller et al. 2018). Recent work suggests that oscillatory activity in the brain may be a form of low-energy analog computing that supports working memory (Lundqvist et al. 2023). Yet modern large-scale AI models, including LLMs, operate with fundamentally static or token-synchronous dynamics. Bridging this gap presents a compelling research frontier: introducing traveling-wave-like dynamics and oscillatory gating into LLMs and other large-scale architectures (Muller et al. 2024). This approach could involve rhythm-based attention windows (Tiesinga et al. 2004; Tiesinga and Sejnowski 2010; Fries 2023), phase-coupled memory activation (Roehri et al. 2022), and dynamic routing paths that emulate the selective coherence seen in cortical circuits (Fries 2023; Banaie Boroujeni and Womelsdorf 2023). Such structured dynamics would not only reduce inference cost and improve temporal coherence but also pave the way for closed-loop agentic systems, where internal wave states regulate external actions in response to changing sensory or goal contexts. Simulating these wave phenomena in neuromorphic or FPGA-based edge architectures can further support real-time, biologically grounded inference under strict energy constraints.

### Synthetic nervous systems

A new neural network architecture, Synthetic Nervous Systems (SNS), has begun to be developed that is more directly inspired by biological nervous systems, requires much smaller training sets, and because of the sparsity of connections, is likely to be far more energy efficient. In the common instantiation, model neurons do not spike, but they can contain multiple ionic conductances, endowing them with complex temporal dynamics similar to those of biological neurons, and their synaptic connections are also dynamic and can change with experience (Szczecinski et al. 2017a). The SNS framework has been extended to simple spiking systems as well (Szczecinski et al. 2020). By using the functional sub-network approach, it is possible to reliably and automatically map a particular function to a small network of these model neurons (Szczecinski et al. 2017b). Once the dynamics of the network have been established, improving parameters using standard optimization techniques is very effective. A software tool allows SNS networks with thousands of neuron to be designed and simulated in real-time (Nourse et al. 2023). These networks have been used for modeling neuromechanical systems, and for the control of biologically-inspired robots.

### Leaner training algorithms should save energy

One of the major differences between natural (human/biological) and artificial intelligence is the ability to (1) learn with few examples, (2) learn continuously with minimal teaching or supervision, and (3) use common-sense reasoning (Choi 2022). These well-documented features save time and energy. Due to availability and relatively low cost of computing resources, current AI systems do not typically attempt to address this need for enormous training datasets, and using data generated by AI systems to train themselves has proven dangerous (Shumailov et al. 2024). The industry has not yet shown a motivation to develop methods to minimize the training sets. New research specifically targeted at continuous learning and making the most of small training datasets techniques and theories (Perera-Lago et al. 2024) could be a significant step forward for energy efficient AI, especially at the “edge” (i.e. close to the user). Alternative algorithms to those commonly used in LLMs today could also be tested for efficiency, as well as superior outcomes (Grossberg 2020; Wang et al. 2024).

### Organoid computation

Investigating the limits of single neuron information processing capabilities has taken many forms over decades in living models from protists such as slime molds to mammalian neurons (e.g., (Zhu et al. 2018; Fitch 2021)). More recently, the organoid, or brain-in-a-dish, approach is showing some promise where small groups of neurons are harnessed to compute input-output relationships, even to the point of playing video games (Kagan et al. 2022). These efforts, and the ability to “design” neurons using technologies such as CRISPR/Cas9, will likely also lead to more synthetic biological approaches to discover multi-scale computation principles (Kagan et al. 2023).

### Offline and offsite processing save time and energy

Our brain processes information during sleep, with minimal energy expenditure. It is also capable of dynamically allocating resources (e.g. attention) to specific tasks or sensory pathways in a context-dependent manner (Murai and Goto 2025). AI is essentially stimulus driven (prompt, get an answer), and is active when humans are active (peak energy expenditure). One could imagine world-wide AI architectures that allocate AI tasks to regions of the world where energy is the cheapest/demand is lowest (e.g. prompt during the day in the USA, get an immediate AI answer/processing at night, in India). One could also imagine AI systems that compile answers, restructure knowledge, and improve their database during “off-line” hours (when humans sleep). Not all AI computations need be human-stimulus driven.

## Discussion

### Longer term concerns

It is time to clarify what we want from AI systems and what AI actually means. Large Language Models (LLMs) are remarkable tools in their early stages but arguably not intelligent systems by any useful definition and their use to achieve Artificial General Intelligence (AGI) is increasingly in doubt (Hofkirchner 2023; Madabushi et al. 2025; Mumuni and Mumuni 2025). LLMs are structurally very limited, being simply rapid statistical sampling and prediction programs that operate on input data. In addition, the efficacy of representing higher-level human cognition with probabilistic models has been questioned (e.g. (Marcus and Davis 2013)). The well-known “hallucinations” (or confabulations) LLMs can produce result from intrinsic design choices that could be conceived as core to finding creative solutions to hard problems, but could also be the result of the processing of bad or incomplete data and inadequate algorithms (Farquhar et al. 2024; Ji et al. 2023). Furthermore, most current AI approaches are difficult to scale. AI technology is already hitting the wall of exponential energy use for very incremental gains in function. The costs of AI are already “obscene” as commented by the New Yorker^2^, elaborated by the MIT Tech Review^3^, and data centers are taking ever more from the grid (de Vries 2023). It costs about $700,000 per day in energy to run ChatGPT 3.5^4^. This seems to us a questionable return on investment. The AI progress envisioned by the “Agent 4” superintelligence in the popular sci-fi speculation based on AI-2027 (https://ai-2027.com/) is very likely physically impossible with current chip architectures, on energy consumption grounds alone.

### Novel mechanisms for research partnerships with industry and/or academia

AI can mimic and eventually improve natural, biological intelligence, the functions and efficiencies of which are far from understood. It therefore stands to reason that we need to understand more about biological intelligence, otherwise AI efforts will be searching for solutions in the dark, forced to implement untested strategies at potentially high financial, energy, and natural resource costs. One problem with the current scientific approach to AI is the limited cross-pollination of ideas between the architects of AI and neuroscientists. On the one hand, most computer scientists and physicists, including those enjoying notoriety in the AI field today, have limited appreciation of neurobiology or cognitive neuroscience. On the other hand, most neurobiologists lack sufficient understanding of computer algorithms, coding and the physics of information processing to contribute to AI technology development. Although there have been some efforts to integrate this knowledge (Hassabis et al. 2017; Botvinick et al. 2020), communication between these research communities remains hamstrung, partially due to siloed training experiences.

Improving and evolving AI will require establishing new research ecosystems where these two approaches to intelligence are encouraged to flourish synergistically, with a new generation of experts who are well-versed in biology, psychology, cognitive science, physics, engineering and computer science. Such integrated cross-disciplinary training should be reflected in new departments at universities, in new funding for multi-disciplinary training at multiple career stages, as well as in initiatives to bridge academic and industry priorities. In contrast to today, 60 years ago, there were no undergraduate neuroscience departments or programs; interested students had to choose between psychology or biology or computer science. It is not unprecedented therefore that the organization of academia should adapt to the new requirements of the society. This process has already started at a few institutions, such as at Rice University^5^, which now offer AI majors, but these curricula still fall short, particularly in biology. Given the challenges facing the research budget of the US government, the integrated involvement of foundations, non-profits and private industry would allow more efficient use of taxpayer allocated resources by reducing overhead costs, enabling administrative agility and creating a profit-sharing environment that fosters innovation.

### Biologically-inspired intelligent devices

In the longer-term, it may be very valuable to consider the contrast between engineered systems and evolution-driven biological systems for generating novel efficient intelligent devices. In general, engineered devices are carefully designed, are usually functionally decomposable for ease of design, maintenance and repair, are manufactured to be identical, and are often controlled using techniques that minimize nonlinearities and maximize ease of predictability. In contrast, biologically systems are subject to *evolutionary processes*, in which individuals vary, and that variation may be essential for survival in an unpredictable and changing environment. The process of *development* organizes an exponentially increasing number of elements in polynomial time into organs and a functioning organism. During the lifespan of an organism, local plasticity rules allow for continual adjustments to a changing environment, allowing organisms to find regularities and “common sense” rules about how the world works through experience. All of these processes are constrained by the need to be as energy efficient as possible, and thus all may provide valuable lessons for creating novel energy efficient autonomous intelligent devices in the future.

### Efficiency and hardware

An important consideration for incorporating biological features into engineered systems is that energy efficiency is controlled by both software and hardware. Many of the mechanisms described in this work, such as event-based communication and sparse computation, would have a minimal benefit on current AI hardware (GPUs/TPUs) which is not designed with those paradigms in mind. Current AI and ML systems have been optimized for these power-hungry platforms as they are the current leaders in the “hardware lottery” (Hooker 2020). Truly energy-efficient AI may require significant investment into developing hardware which is designed from the beginning to harness these biologically-grounded mechanisms of energy-efficiency and intelligence.

## Data Availability

No datasets were generated or analysed during the current study.

## Footnotes

1 https://www.geeksforgeeks.org/deep-learning/tanh-vs-sigmoid-vs-relu/

2 https://www.newyorker.com/news/daily-comment/the-obscene-energy-demands-of-ai

3 https://www.technologyreview.com/2025/05/20/1116327/ai-energy-usage-climate-footprint-big-tech/

4 https://www.universityofcalifornia.edu/news/researchers-run-high-performing-large-language-model-energy-needed-power-lightbulb

5 https://news.rice.edu/news/2025/rice-offer-bachelor-science-artificial-intelligence
